# Long COVID incidence across SARS-CoV-2 lineages and identification of conserved spike targets for multivalent vaccines

**DOI:** 10.1017/cts.2025.10226

**Published:** 2025-12-19

**Authors:** Grace Jaeyoon Kim, Md Ashad Alam, Judy S. Crabtree, Rebecca Rose, Susanna L. Lamers, San Chu, Ronald Horswell, Daniel Fort, Lucio Miele

**Affiliations:** 1 Department of Genetics, Louisiana State University Health Sciences Center, New Orleans, LA, USA; 2 School of Medicine, Louisiana State University Health Sciences Center, New Orleans, LA, USA; 3 Ochsner Center for Outcomes Research, Ochsner Medical Center, New Orleans, LA, USA; 4 Department of Research and Development, BioInfoExperts LLC, Thibodaux, LA, USA; 5 Department of Population and Public Health, LSU Pennington Biomedical Research Center, Baton Rouge, LA, USA; 6 LSU-LCMC Health Cancer Center, New Orleans, LA, USA

**Keywords:** Covid-19, long covid, PASC, electronic medical records, translational medicine

## Abstract

**Background:**

Long COVID remains poorly characterized at the genomic level. The primary aim of this study was to examine the relationship between viral sequences and the incidence of Long COVID at a tertiary care center in Louisiana between April 2020 and December 2022. A secondary aim was analysis of the Spike protein to identify conserved regions for multivalent vaccine targets.

**Method:**

To estimate Long COVID incidence across variants, we linked 4789 SARS-CoV-2 sequences to 3090 de-identified patient electronic health record information. The base population was defined as any patient with an International Classification of Diseases-10-Clinical Modification COVID-19 diagnosis code (U07.1) based definitions of Long COVID presentation developed by the N3C consortium.

**Results:**

1,554 patients (1,536 Long COVID-negative) met Long COVID definitions, with 56.3% being female, 36.1% self-reported as African American, 5.5% self-reported as Hispanic/Latino, and 54.5% had received at least one vaccine dose 14 days prior to SARS-CoV-2 collection. Long COVID-positive patients were older (mean age 43.1 years) than negative patients (35.9 years; *p* = 0.0054) and were more likely to be female (*p* = 0.0001). Among unvaccinated patients, those with Long COVID were significantly younger than their vaccinated counterparts (*p* < 0.00001). Long COVID incidence varied by PANGO lineage, ranging between 14% in AY.13 to 67.8% in B.1.1.7. Analysis of spike protein diversity revealed eight conserved amino acid regions (Shannon entropy < 0.43), representing potential targets for vaccine design.

**Conclusion:**

Long COVID rates across thousands of annotated SARS-CoV-2 sequences revealed lineage-specific risk and conserved epitopes for future interventions.

## Introduction

Unlike the typical clinical spectrum of acute infection, postacute sequelae of severe acute respiratory syndrome coronavirus 2 (SARS-CoV-2) infection (PASC) encompasses heterogeneous symptoms affecting one or more organ systems [1]. As it is currently defined, PASC, also known as Long COVID, is the ongoing, relapsing, or development of new symptoms/conditions present for more than 3 months following SARS-CoV-2 infection, regardless of recognition [1,2]. Prevalence estimates vary widely, ranging from “10 to 35 percent or higher” [2–6]. This broad range reflects the evolving definitions of the disease and “potentially overlapping etiologies,” such as autoimmunity, clotting and endothelial abnormalities, microbiota dysregulation, and immune dysfunction [1,2,7]. Disease presentations show substantial diversity with several distinct “clusters,” including cardiac, pulmonary, neurological, pediatric/gastrointestinal, and metabolic/obesity related complications [8–10]. While men are at greater risk of experiencing severe acute COVID-19, the female sex is more likely to develop Long COVID [1,7,8,10–12]. Although the relationship between SARS-CoV-2 vaccination and Long COVID requires further elucidation, emerging evidence suggests that prior immunization may confer a reduced risk of Long COVID outcomes [8,13–16].

SARS-CoV-2 variants and subvariants may affect clinically relevant characteristics such as risks of reinfection [17,18], protection against neutralizing antibodies [19–21], viral transmissibility [22], disease severity [22,23], and Long COVID risk [10,17,24,25]. Several studies, including the NIH-sponsored “Researching COVID to Enhance Recovery” (RECOVER) reports, suggest initial infections, particularly with Delta variants, have greater incidence of Long COVID [10,17,24,26]. However, these associations are limited due to the imprecise extrapolation of COVID-19 variant epochs via SARS-CoV-2 diagnostic testing dates. As New Orleans was an early COVID-19 hotspot, we sequenced the genomes of SARS-CoV-2 from patients positive for COVID-19 between April 2020 and December 2022. This allowed us to match 3090 de-identified health records within the Ochsner Health System to viral sequences. Therefore, the primary objective of this study was to examine whether SARS-CoV-2 viral sequences correlate to differences in Long COVID rate based upon EHR data from each sample’s infected host. While prior work has reported differential Long COVID rates by viral variant, basic demography, or examined characteristics of single consensus samples, to our knowledge this is the first effort to examine rates of Long COVID across thousands of patients with individual SARS-CoV-2 viral sequences. The secondary objective of this study was an examination of the variability of the amino acid sequence of the SARS-CoV-2 spike protein to assess whether variations in sequence had similar associations with Long COVID as the underlying DNA. Amino acid sequences which are conserved across multiple variants could inform multivalent vaccine development.

## Materials and methods

### SARS-CoV-2 viral genome sequencing

Specimens were received and accessioned from various Ochsner collection sites, covered by LSUHSC-NO IRB #1440 and Ochsner IRB # 2021.221 - LDH Variant outcomes. RNA extraction and sequencing methodology, as previously described [27].

### Base population and long COVID definition

Electronic health record data were queried for subjects with a positive SARS-CoV-2 sample with viral sequencing (Ochsner IRB # 2022.326, Pennington IRB # 2021-038-PBRC NIGMS, Pennington IRB # 2023-019-PBRC). For each subject’s full data window (from –730 to + 270 from sample collection), we extracted the following: patient identifier, demographics, a “refdate” (date of specimen collection), age at “refdate,” viral genomic data, COVID-19 vaccination records, outpatient encounter data, and clinical diagnosis codes. Vaccination was defined as having received one dose at least 14 days before SARS-CoV-2 sample collection. A subject was classified as having Long COVID if their record contained any ICD-10-CM code consistent with the N3C-derived Long COVID phenotype that first appeared on or after day 90 following “refdate” and persisted or recurred through at least day 270.

### Odds ratio calculation

We used adjusted odds ratios (aORs) to compare Long COVID incidence among each PANGO variant’s cases to Long COVID incidence among all other variants combined. Odds ratios were adjusted for race/ethnicity (black, white, Hispanic), gender, and age (10-year age groups). In estimating the aOR for each target variant, the combination of other variants was weighted to the demographic composition of the target variant’s cases.

### Spike amino acid conservation score analysis

We conducted a binomial proportion test for PANGO variants with more than five sequences. This test is associated with the analysis of viral variants, potentially within the framework of the PANGO lineage classification system for categorizing SARS-CoV-2 variants. Based on the resulting *p*-values, we can infer that there is a significant distinction among the PANGO Variants. Furthermore, apart from the PANGO variants analysis, we have conducted an examination of the amino acids within the spike protein. To achieve this, we have generated a conservation score plot of amino acids which enables us to visualize the extent of variation at each position along the protein sequence regions with peaks often correspond to functional domains or important binding sites within the protein.

### IEDB tepitools in silico CD8+ T cell analysis

Variant specific spike, membrane, and nucleocapsid cDNA sequences were generated from Ensembl’s SARS-CoV-2 genome browser (RRID:SCR_024704) and the Expasy translate tool (RRID:SCR_024703). We computationally determined binding predictions of MHC Class I SARS-CoV-2 epitopes using the Immune Epitope Database and Analysis (IEDB) Resource TepiTool, utilizing the IEDB recommended default prediction for a panel of 27 most frequent *A* and *B* alleles: HLA-A*01:01, −A*02:01, −A*02:03, −A*02:06, −A*03:01, −A*11:01, −A*23:01, −A*24:02, −A*26:01, −A*30:01, −A*30:02, −A*31:01, A*32:01, −A*33:01, −A*68:01, −A*68:02, −B*07:02, B*08:01, −B*15:01, −B*35:01, −B*40:01, −B*44:02, −B*44:03, −B*51:01, −B*53:01, −B*57:01, and −B*58:01. IEDB’s default prediction method reflects consensus across ANN, SMM and CombLib predictors and was used to select peptides with predicted consensus percentile ranks ≤ 1 (35) Concise results between variant-specific protein products were analyzed on *R* using the packages readxl and dplyr.

## Results

### Cohort description

The full characteristics of study participants can be found within the Supplementary Materials (Supplementary material 1). The study population was defined as any patient with an International Classification of Diseases-10-Clinical Modification COVID-19 diagnosis code (U07.1) based definition of Long COVID presentation developed by the N3C consortium [1] and further refined in-house. A total of 3090 patients (Table 1), 1554 Long COVID positive; 56.3% female [1739/3090]; 36.1% self-reported African American [1118/3090]; 5.5% Hispanic/Latino [170/3090]; 54.5% vaccinated; average age 39.5 years, standard deviation (SD) 20.2) and 4,789 viral sequences were included in this study. We used the Ochsner Health database to build a study population of 1,554 (50.29%) adults with Long COVID demographically matched by age, sex, and self-reported race to 1,536 (49.71%) acutely infected patients without Long COVID development. With respect to vaccination status, 54% of included patients received at least one COVID-19 vaccination (48.6% 2 or more doses, 5.9% or 181/3090 1 dose) (Table S1 in Supplementary material 1). As an N3C trusted vaccination site (24), we have high confidence in the accuracy and reliability of our vaccination data.

**Table 1.** Patient demographics, a total of 3090 patients (1554 long COVID positive, 1536 long COVID negative) were included in this study. Long COVID patients were more likely to be older in age (43 years vs 36 years, *p* = 0.0054) and of female sex (52.9% women [920/1739]; 40.41% men [634/1351]; *p* value = 0.0011)

			Long COVID	
Demographics		Total	Yes	No
Overall		3090 (100)	1554 (50.29)	1536 (49.71)
Age		39.53 ± 20.24	43.09 ± 20.30	35.94 ± 19.52
Gender	Female	1739 (56.28)	920 (52.90)	819 (47.10)
	Male	1351 (43.72)	634 (40.41)	717 (54.59)
Race	Native	15 (0.49)	9 (60.00)	6 (40.00)
	Asian	41 (1.33)	15 (36.59)	26 (63.41)
	African American	1118 (36.11)	217 (47.14)	591(52.86)
	Other	44 (1.43)	16 (26.36)	28 (63.64)
	Refused	14 (0.22)	3 (21.43)	11 (78.57)
	Unknown	44 (1.43)	2 (4.55)	42 (95.45)
	White	1814 (58.71)	982 (54.13)	832 (45.87)
Ethnicity	Hispanic	170 (5.50)	87 (51.18)	83 (48.82)
	Not Hispanic	2840 (91.91)	1460 (51.41)	1380 (48.59)
	Refused	19 (0.62)	4 (21.05)	15 (78.95)
	Unknown	61 (1.97)	3 (4.92)	58 (95.08)
Vaccinated	Vaccinated	1683 (54)	1007 (59.8)	676 (40.2)
	Female	1041 (61.9)	631 (60.6)	410 (39.4)
	Male	642 (38.1)	376 (58.6)	266 (41.4)
	Female age	45.36 ± 18.76	46.99 ± 18.87	42.86 ± 18.34
	Male age	46.68 ± 18.9	49.56 ± 18.09	42.61 ± 19.21
Unvaccinated	Not vaccinated	1407 (45.5)	547 (38.9)	860 (61.1)
	Female	698 (49.6)	289 (41.4)	409 (58.6)
	Male	709 (50.4)	258 (36.4)	451 (63.6)
	Female age	32.51 ± 19.31	35.18 ± 19.88	30.62 ± 18.70
	Male age	31.42 ± 19.2	32.97 ± 20.67	30.54 ± 18.31

Patients experiencing Long COVID were statistically more likely to be of the female sex (table S1 in Supplementary material 1, aOR, 1.317, 95% CI 1.145 to 1.514) and older in age (Table 1, Long COVID-positive: 43.09 ± 20.30 years, Long COVID negative: 35.94 ± 19.52, *p* = 0.0054) (table S1 in Supplementary material 1, aOR, 1.947; 95% confidence interval [CI], 1.523 to 2.489), regardless of vaccination status (table S2 in Supplementary material 1, vaccinated Long COVID: 47.95 ± 18.61, unvaccinated Long COVID: 42.76 ± 18.67). Female Long COVID patients were statistically older than those without, independent of vaccination status (table S2 in Supplementary material 1, vaccinated females *p* = 0.0005, unvaccinated females *p* = 0.0021). In contrast, age did not significantly differ between unvaccinated men with or without Long COVID, while vaccinated men with Long COVID were significantly older than their counterparts who did not develop the condition (table S2 in Supplementary material 1, 49.56 vs 42.61, *p* < 0.0001).

### Long COVID incidence by PANGO variant

**Figure 1.**
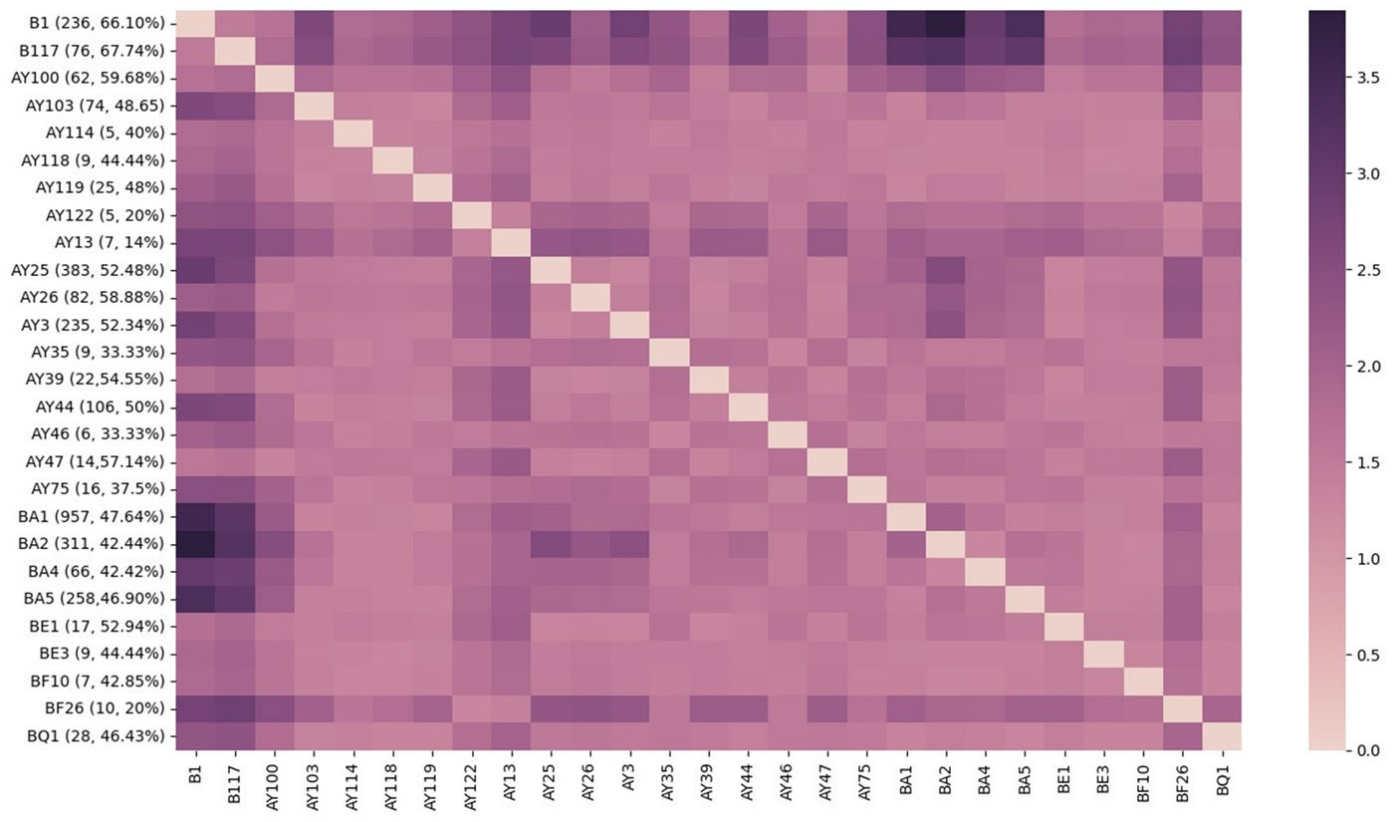
Incidence of long COVID by PANGO variant. Proportion of viral variants (labeled as [variant, *n*_patients infected with variant_ =, proportion developing long COVID], ex. B.1 [236, 66.1%]) resulting in long COVID plotted against inverse log Benjamini–Hochberg adjusted *p*-values (≥1.3 indicates significance). SARS-CoV-2 variants are listed in chronological order of appearance from earliest (ancestral B.1) to most recent (Omicron BQ.1), with a sample size less than *n* = 5 were excluded from this analysis. This figure was generated using the Seaborn python data visualization library.

We conducted a Binomial proportion test for Long COVID incidence by SARS-CoV-2 subtypes, using dynamic nomenclature, for 27 variants found in 3090 de-identified patients within the Ochsner Health System. While rates of Long COVID ranged between 14% (AY.13; *n* = 1/7) and 67.8% (B.1.1.7; *n* = 51/76), most variants reported an incidence between 40% and 53% (Figure 1). Rates remained high throughout the pandemic, although we observed a declining trend characterized by a reduction in the proportion of individuals affected by Long COVID as successive SARS-CoV-2 variants emerged (Figure 1).

**Figure 2.**
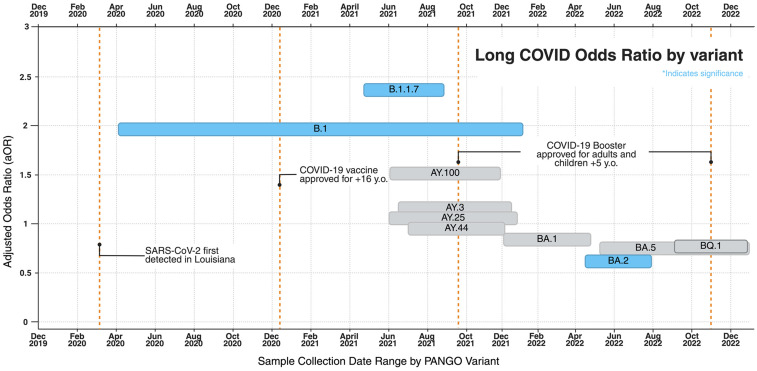
Adjusted odds ratios of long COVID by variant group plotted by sample collection dates. Odds ratios are adjusted, via stratification, for age, gender, and race and reflect values indicated in Table S3. Statistical significance is indicated by the blue coloring.

Compared to other variants, patients infected with pre-alpha B.1 (*n* = 236) had 1.955 greater adjusted odds (95% CI, 1.359 to 2.812; *p* value = 3.05 × 10^–4^) of developing Long COVID (table S3 in Supplementary material 1). The likelihood of developing Long COVID was greatest in Alpha variant, B.1.1.7 (*n* = 75; OR = 2.396; 95% CI, 1.229 to 4.672; *p* = 0.010) and smallest in Omicron BA.2 (*n* = 309; OR = 0.599; 95% CI, 0.436 to 0.824; *p* = 0.002) (Figure 2, table S3 in Supplementary material 1).

### Vaccination status of patient cohorts

Of the 3,090 patients included in this study, 10,683 (54.5%) were classified as vaccinated, defined in this study as having received at least one COVID-19 vaccine dose at least 14 days before a positive SARS-CoV-2 sample collection (Table 1, table S2 in Supplementary material 1). Vaccinated patients were significantly older (table S2 in Supplementary material 1, 45.87 years vs 31.96 years “not vaccinated,” *p* < 0.0001) and significantly more likely to be female (Table 1, *p* < 0.0001). Additionally, vaccinated patients had significantly greater representation of participants over 65 years old (17.6% vs 5.8%, *p* < 0.0001) and women over 50 years (26.2% vs 10.7%, *p* < 0.0001) (table S2 in Supplementary material 1). Unvaccinated individuals who developed Long COVID were significantly younger than vaccinated Long COVID patients (Women: 35.2 ± 19.9 vs 46.9 ± 18.9 years; Men 33.0 ± 20.67 vs 49.6 ± 18.1 years) (table S2 in Supplementary material 1). Among patients infected with pre-alpha and alpha variants, vaccination was rare (3% in B.1 and 17% in B.1.1.7), and those immunized were older than unvaccinated counterparts (B.1: 60.9 vs 40.2 years; B.1.1.7: 50.9 vs 33.9 years) (table S2 in Supplementary material 1).

### Epitope differences in variants associated with differential SARS-CoV-2 risk

Previously published work by our group [27] analyzed CD8+ epitope diversity for 27 common HLA-A and -B alleles across the ancestral Wuhan strain (NCBI: NC_045512.2) and 16 SARS-CoV-2 variants sequenced from the Louisiana patient population. Predicted MHC Class I epitopes of spike proteins from emerging SARS-CoV-2 variants were compared to those of the original Wuhan strain, the basis of the initial COVID-19 vaccine, using the Immune Epitope Database (IEDB) TepiTool. Of the total 1,115 CD8+ spike epitopes, roughly 72.4% (807) were conserved across all 17 variants (Figures S2, S3, S4 in Supplementary material 1 and Supplementary material 2). Emerging variants had 1% (B.1) to 89% (XBB.1) of spike epitopes experiencing putative alterations in predicted binding when compared to ancestral Wuhan epitopes (B.1.1.7 47.4% [519/10950], AY.25 8.9% [97/1084], AY.3 8.4% [90/1077], BA.2 38.3% [422/1102]) (fig. S4 in Supplementary material 1). Despite the higher incidence of Long COVID in PANGO variants B.1 and B0.1.1.7 within the Ochsner Health System, only 96 unique Spike epitopes were identified in B.1.1.7, with 24 distinct peptide sequences. The peptide QSYGFQPTY affected the largest number of MHC Class I alleles (8/16 HLA-A, 5/11 HLA-B) (Supplementary material 2).

### Conserved and accessible spike targets proposed for vaccine development

**Figure 3.**
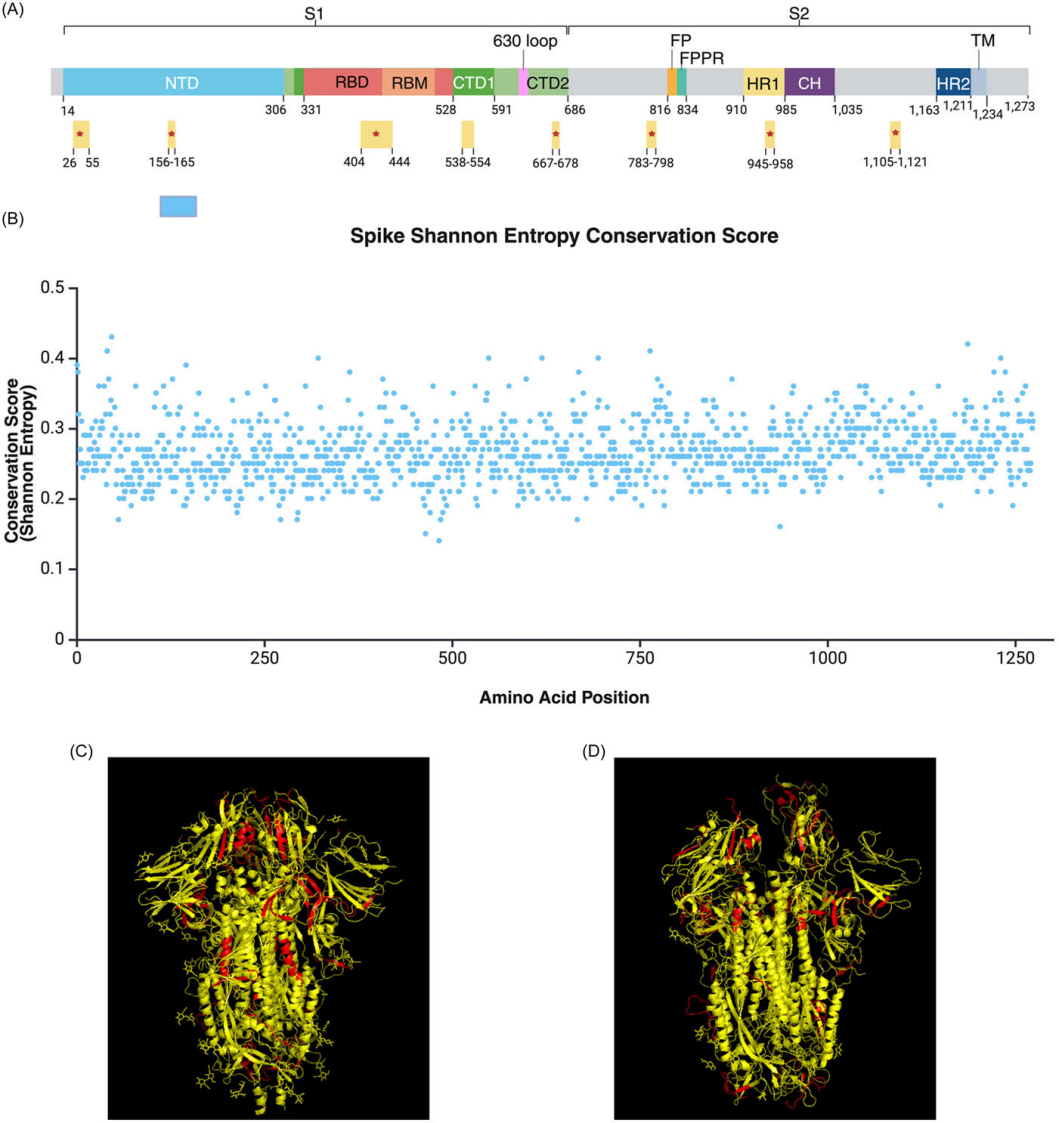
Conserved and accessible regions of the SARS-CoV-2 spike protein proposed for multivalent vaccine development. (A) Highly conserved and accessible regions (*N* = 8) displayed by spike protein domains. *Indicates accessibility in both open and closed confirmations. Figure 3A was adapted from Jackson et al. 2022 [34] (**B**) shannon entropy conservation score of spike amino acid positions (*N* = 1,273). Values range from 0 to 1, with higher values indicating greater probability of amino acid conservation. All positions had a value < 0.43, reflecting the high mutability of the spike protein. Conservation scores can be found in table format within the supplemental data files. Figure 2A-B was generated using biorender. (**C** to **D**) Ribbon structure of conserved regions, highlighted in red (*N* = 8), viewed in closed (PDB: 6VXX) (**C**) and open (PDB: 7ZH5) (**D**) protein confirmations using PyMOL.

To examine the diversity of SARS-CoV-2 spike sequences, we calculated Shannon Entropy conservation scores for the 4,789 variant spike sequences analyzed in this study. All 1273 amino acid positions had conservation scores below 0.43, reflecting the high mutability of the Spike protein (Figure 3B, figure S2 in Supplementary material 1). Still, we identified several conserved regions spanning spike protein domains such as the N-terminal domain (NTD), receptor binding domain (RBD), receptor binding motif (RBM), cytoplasmic tails 1 and 2 (CTD1 and CTD2), and heptad-repeat domain 1 (HR1) (Figure 3A, Supplementary material 3). Generated Shannon entry conservation scores were combined with published accessibility scores for spike protein residues (27) to determine potential targets for multivalent vaccines. Of the proposed regions, spike residues 26–55, 156–165, 404–444, 667–678, 783–798, 945–958, and 1,105–1,121 are accessible in both closed (Figure 3C) and open (Figure 3D) protein confirmations.

127 (15.7%) of the 807 conserved CD8+ spike epitopes can be found in the proposed regions (Supplementary material 2 and 3). The conserved epitopes comprise of 37 unique peptides affecting all 16 HLA-A and 8/11 HLA-B alleles analyzed, excluding HLA-B*40:01, -B*44:02, and -B*44:03 (Supplementary material 3). Of these epitopes, STQDLFLPF (11/27 HLA alleles), NSFTRGVYY (8/27), SANNCTFEY (7/27), KVFRSSVLH (6/27), GTHWFVTQR (6/27) and NTQEVFAQV (6/27) have maximal HLA Class I allelic coverage (Supplementary material 2).

## Discussion

The primary objective of this study was to examine whether differences in COVID-19 viral sequence correlate to differences in Long COVID rates derived from EHR data from each sample’s infected host. In this study involving 3,090 patients with SARS-CoV-2 infection and 4,789 viral sequences, Long COVID rates typically ranged between 40% and 53%, with outliers in the Ancestral B.1 (66.1%; OR: 1.955; 95% CI, 1.359 to 2.812), Alpha B.1.1.7 (67.7%; OR: 2.396; 95% CI, 1.229 to 4.672) and Omicron BA.2 (42.44%; OR: 0.599; 95% CI, 0.436 to 0.824) variants (Figure 1, table S3 in Supplementary material 1). The number of persons experiencing Long COVID remained high throughout different eras of the pandemic, with affected persons being statistically more likely to be older in age (Table 1, *p* = 0.0054) (table S1 in Supplementary material 1, aOR, 1.947; 95% CI, 1.523 to 2.489) and of female sex (Table 1, *p* = 0.0011) (table S1 in Supplementary material 1, aOR, 1.317, 95% CI 1.145 to 1.514). Assuming that pre-existing immunity is protective against Long COVID, it is reasonable to expect that the earliest variants of a novel virus infecting an immunologically naive population, like Alpha and pre-Delta, are associated with higher incidence of Long COVID. Our findings validate previous reports that suggest incidence of Long COVID is lower in Omicron than in Delta [3,8,10,17,24,25,28,29]. The progressive decline in Long COVID incidence and risk across successive variants (Figure 1, 2, table S3 in Supplementary material 1) may be influenced by the accumulation of population-level immunity, as suggested by other studies [28].

Unvaccinated patients who developed Long COVID were significantly younger than their vaccinated counterparts, with a mean difference of 13.81 years (11.8 and 16.6 years younger in women and men, respectively, *p* < 0.00001, table S2 in Supplementary material 1). Additionally, unvaccinated women who developed Long COVID were significantly older than unvaccinated men with Long COVID (35.18 vs 32.97 years, *p* = 0.0412, table S2 in Supplementary material 1). These findings coupled with the increased incidence of Long COVID in women over 50, a surrogate for post-menopausal age, underscores the need for further analysis into the relationship between hormonal changes in Long COVID risk. Our findings validate the “not negligible… high number of persons with PASC” among vaccinated persons seen throughout COVID-19 eras within the Veterans Affairs Health Care System (22) and others [2,3,8]. Of the 1,683 (54%) patients who received at least one COVID-19 vaccine dose at least 14 days prior to SARS-CoV-2 infection, vaccinated individuals were significantly older (45.87 vs 31.96; *p* < 0.0001) (Table S2 in Supplementary material 1) and more likely to be women (63% [795/1260]; *p* < 0.0001) (Table S2 in Supplementary material 1). Our cohort exhibited significantly higher proportion of participants over 65 years old (17.6% vs 5.8%, *p* < 0.0001) and women over 50 years (26.2% vs 10.7%, *p* < 0.0001) (table S2 in Supplementary material 1) and lower rates of vaccination than those documented in previous studies [3,8]. Because our cohort inherently excludes those whose infection would have been prevented by vaccination, our dataset would be unsuitable for estimating any causal effect of vaccination on Long COVID. Therefore, further research is required to clarify how SARS-CoV-2 variants and immunological exposure influences Long COVID incidence.

Among the 4,789 SARS-CoV-2 spike sequences analyzed, all 1273 amino acid positions had Shannon entropy conservation scores below 0.43, highlighting the high mutability of this surface protein crucial for viral entry (Figure 3B). We observed a limited number of effective domains and conserved sequences shared within and between PANGO lineages, which may suggest repetitive cycles of convergent evolution or “rediscovery” of a limited number of active motifs (figures S2–S3 in Supplementary material 1). Considering the potential protective effects of SARS-CoV-2 vaccination against Long COVID development [3,13–15,30]), a multivalent vaccine targeting our proposed conserved spike targets (Figure 3A). could potentially produce long-term immunity and broader protection against future variants. Our findings suggest that naive or waning immunity, due to short-lived immune responses and viral mutations as well as age, is associated with significantly increased odds of Long COVID with select variants. Considering the protective effects of COVID-19 vaccination against the symptoms and severity of Long COVID [8,30,31], multivalent vaccines targeting conserved spike regions could improve long-term immunity and offer broader protection against future variants. We hope that the findings of our study will encourage the development of vaccines offering broader coverage against SARS-CoV-2.

Our study has several key strengths. We utilized the extensive healthcare databases of Ochsner Health System to incorporate SARS-CoV-2 sequences matched to the infected host’s electronic medical records. Early during the COVID pandemic, Ochsner Health built an automated interface to the Louisiana’s state vaccine registry, ensuring completeness of vaccination records. While prior work has reported differential Long COVID rates by viral variant, basic demography, or examined characteristics of single consensus samples, to our knowledge this is the first effort to examine incidence of Long COVID across thousands of clinically annotated COVID-19 viral sequences. As an early COVID-19 hot spot, we provided incidence estimates for less published variants, such as the ancestral B.1.1.7 and Alpha B.1. Finally, we extended our analysis to examine the association between SARS-CoV-2 spike sequences and Long COVID incidence and recommend several targets for future SARS-CoV-2 polyvalent vaccines.

This study has several limitations, namely Ochsner Health Systems being a single site, representative of patients in Louisiana. These findings will need to be validated in patient populations with different demographics. Additionally, we relied on encounter-level clinical data for diagnoses, so the true incidence of Long COVID may be unobserved, as no routine laboratory test for Long COVID is available. It is important to note that our convenience sample of positive COVID tests, while being representative of those seeking testing in an Urgent or Emergency Care context within Ochsner Health, do not represent the underlying vaccinated and unvaccinated populations. This study is limited to the variant-specific risk of developing Long COVID for vaccinated vs. unvaccinated patients who received a positive COVID test, without considering the ramifications of the demonstrable benefit of reducing COVID infection due to vaccination [32,33]. While our sample size was large enough to demonstrate statistically significant differences based on conserved spike protein motifs, the absolute values of Long COVID rates remained high across the subvariants we identified. This suggests that spike protein genetic variability plays a limited role in modulating the risk of Long COVID, and other regions of the viral genome may contribute to the differences we observed.

## Supporting information

10.1017/cts.2025.10226.sm001 Kim et al. supplementary material 1 Kim et al. supplementary material

10.1017/cts.2025.10226.sm002 Kim et al. supplementary material 2 Kim et al. supplementary material

10.1017/cts.2025.10226.sm003 Kim et al. supplementary material 3 Kim et al. supplementary material

10.1017/cts.2025.10226.sm004 Kim et al. supplementary material 4 Kim et al. supplementary material

10.1017/cts.2025.10226.sm005 Kim et al. supplementary material 5 Kim et al. supplementary material

10.1017/cts.2025.10226.sm006 Kim et al. supplementary material 6 Kim et al. supplementary material

## Supplementary material

The supplementary material for this article can be found at https://doi.org/10.1017/cts.2025.10226.
