# Supplementary material for: Long COVID incidence across SARS-CoV-2 lineages and identification of conserved spike targets for multivalent vaccines
Source: J Clin Transl Sci. 2025 Dec 19;9(1):e288. doi: 10.1017/cts.2025.10226 (PMC12780800; doi:10.1017/cts.2025.10226)
Supplement: Kim et al. supplementary material 2 — Kim et al. supplementary material [file S2059866125102264sup002.docx]

Supplementary Materials for

Long COVID risks by variant: Integrating SARS-CoV-2 viral sequences and patient medical records

Grace J. Kim^1,2^†, Md Ashad Alam^3^†, Judy S. Crabtree^1^, Rebecca Rose^4^, Susanna L. Lamers^4^, San Chu^5^, Ronald Horswell^5^, Daniel Fort^3^‡, and Lucio Miele^1^‡*

Corresponding author: [Lmiele@lsuhsc.edu](mailto:Lmiele@lsuhsc.edu)

**The PDF file includes:**

Materials and Methods

Figs. S1 to S4

Tables S1 to S3

**Other Supplementary Materials for this manuscript include the following:**

Kim et al. Supplementary material 2:

Raw dataset of IEDB generated Spike CD8^+^ T epitopes for 27 common HLA-A and -B alleles across the ancestral Wuhan strain (NCBI: NC_045512.2) and 16 SARS-CoV-2 variants sequenced from the Louisiana patient population ([*28*](#_ENREF_28)).

Kim et al. Supplementary material 3:

Raw data of Shannon conservation scores for 4,789 SARS-CoV-2 Spike sequences.

Kim et al. Supplementary material 4:

Raw dataset for 3090 patients included in this study. Dataset includes Genbank Accession Number, subject gender, self-reported race (labeled “race”), self-reported ethnicity, “Refdate”, vaccine count (labeled “VACC_COUNT”), and Long COVID status. Patients meeting N3C definitions of Long COVID were labeled as 1 (0 indicates no Long COVID).

Kim et al. Supplementary material 5:

Raw dataset for 4,789 Ochsner Health SARS-CoV-2 sequences. “Age” reflects subject age at date of data accession, not at time of infection.

Kim et al. Supplementary material 6:

Counts and average ages of vaccinated and unvaccinated cohorts for PANGO lineages B.1, B.1.1.7, AY.100, AY.103, AY.25, and AY.26.


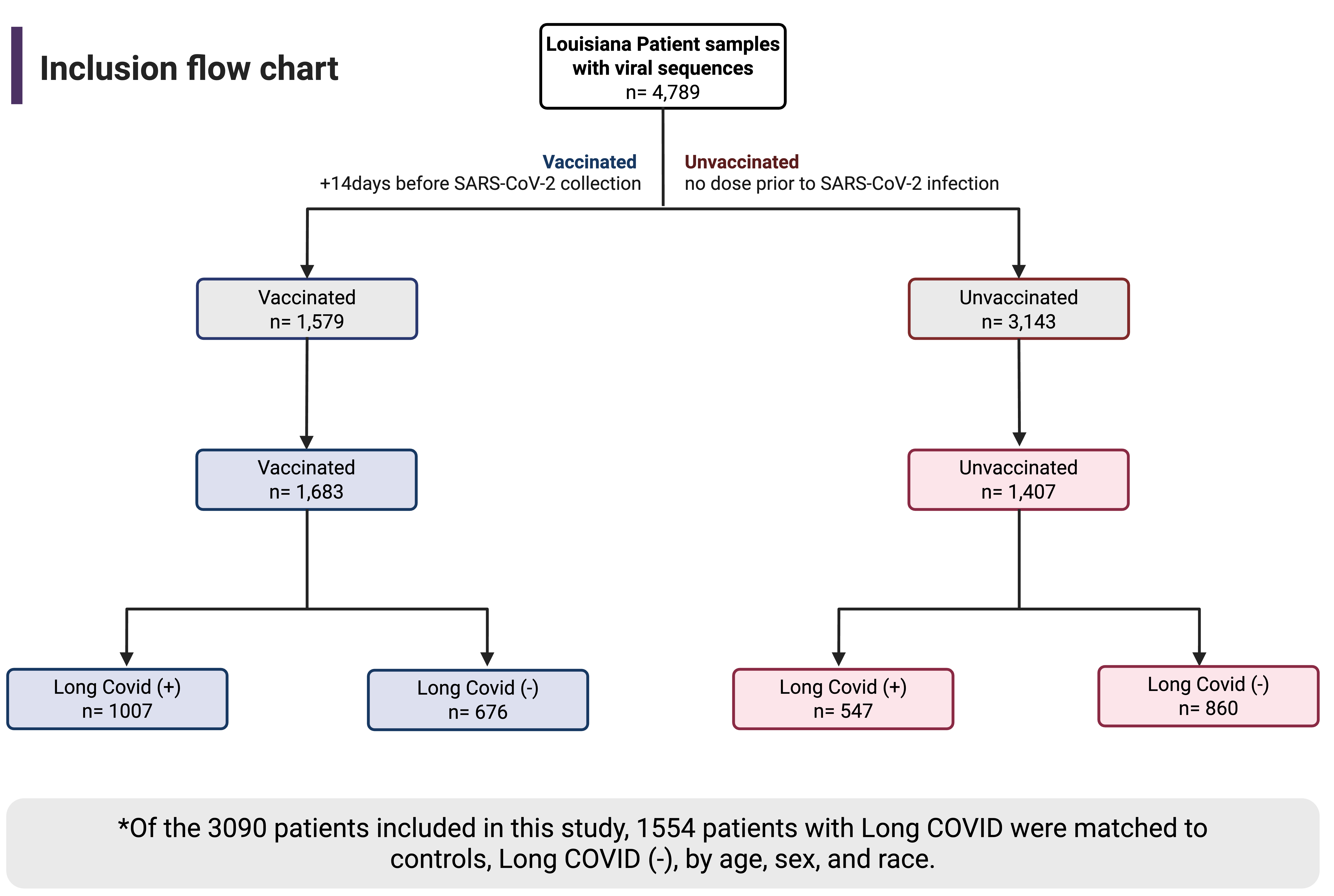


Fig. S1. Patient inclusion flow chart.

Vaccination was defined as having at least one COVID-19 vaccine at least 14 days before a positive SARS-CoV-2 sample collection This figure was generated using BioRender.


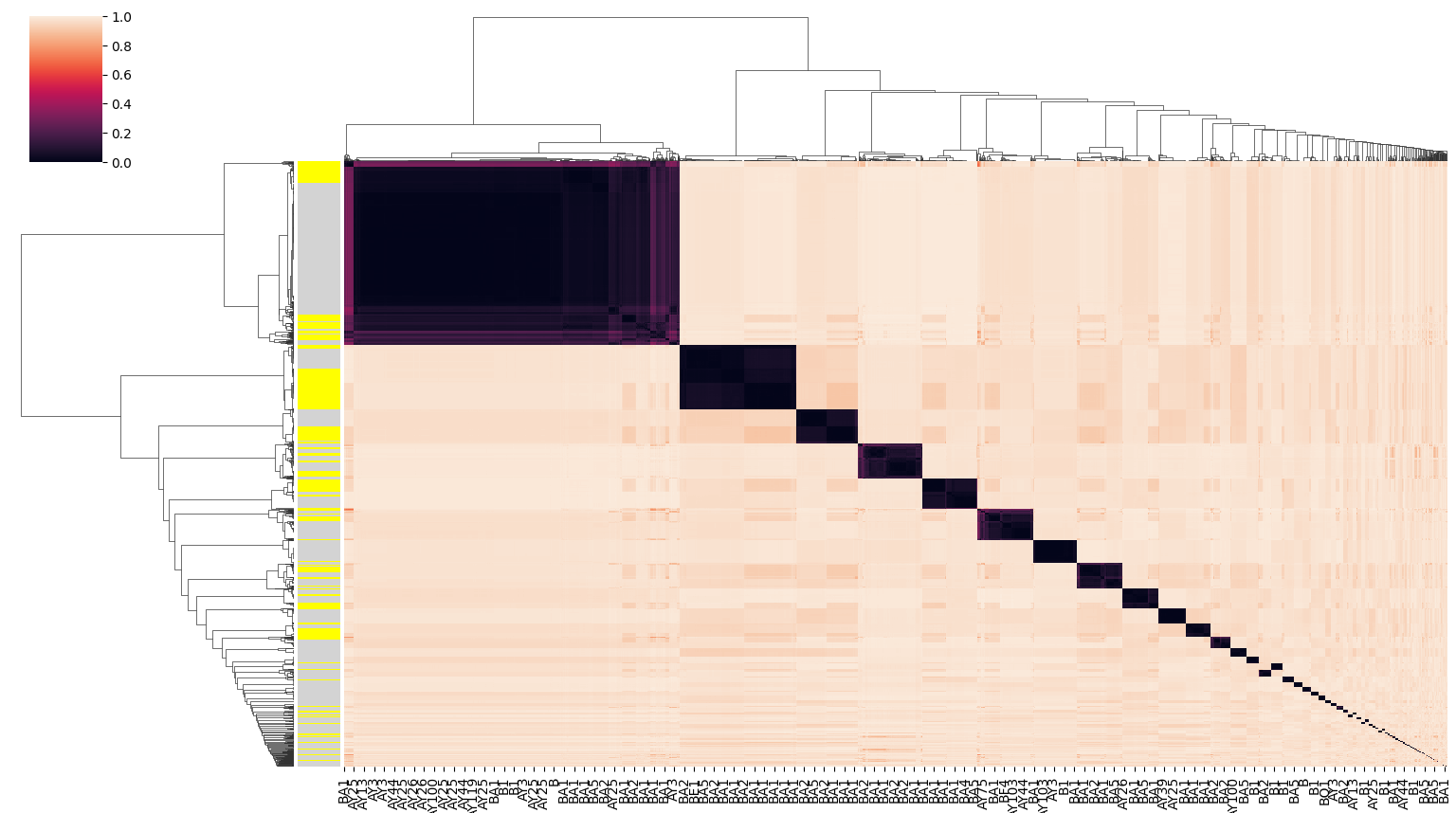
Fig. S2. Hierarchical cluster analysis of 4,789 SARS-CoV-2 sequences.

Hierarchical clustering of spike variants span multiple PANGO lineages and exhibit minimal similarity to each other.


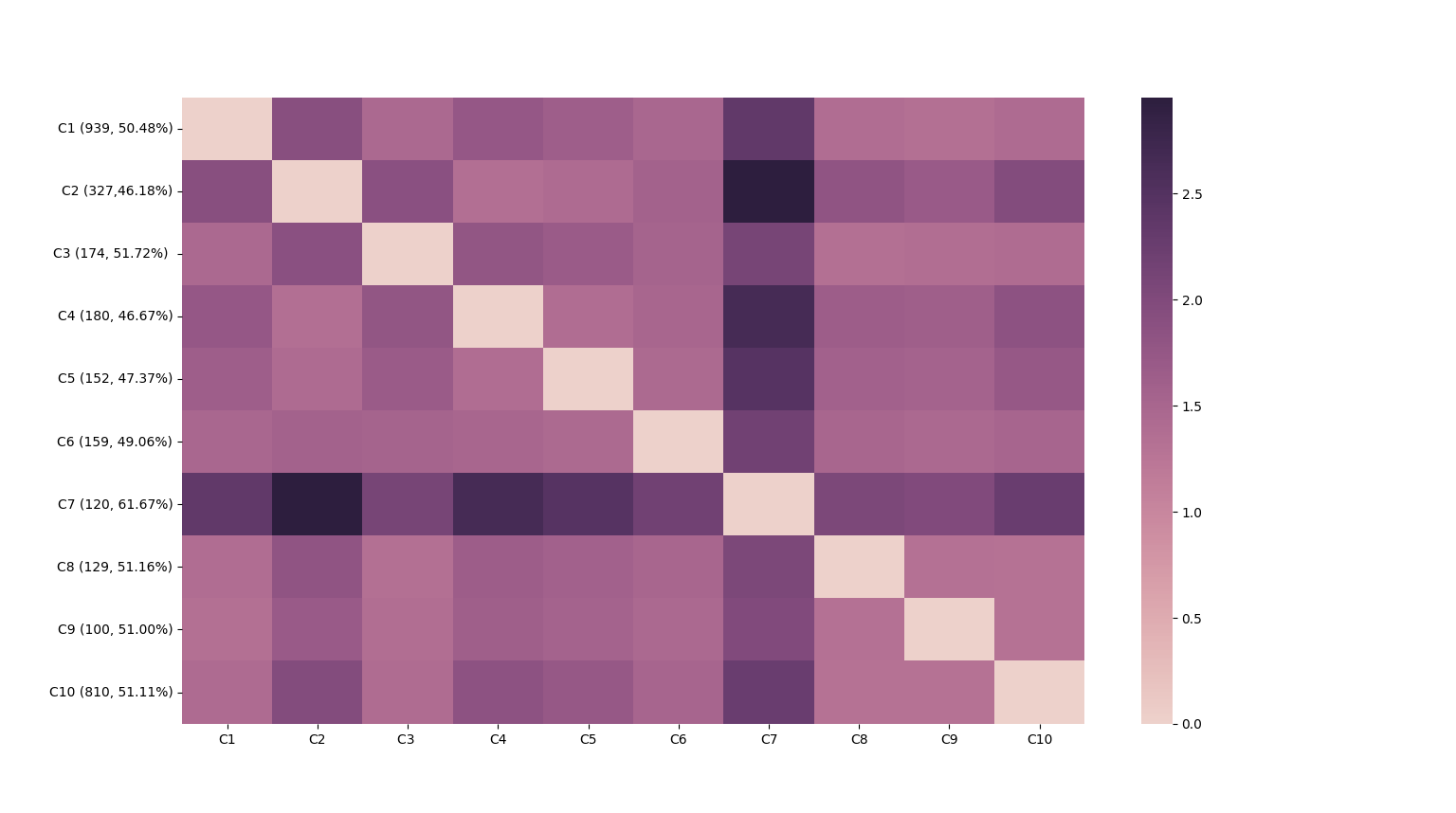


Fig. S3. Incidence by hierarchical clusters.

Rates of Long Covid by hierarchical clusters from Fig. S2 plotted against inverse log Benjamini-Hochberg adjusted p-values comparing clusters detailed in Fig S2 against all others (≥ 1.3 indicates significance).


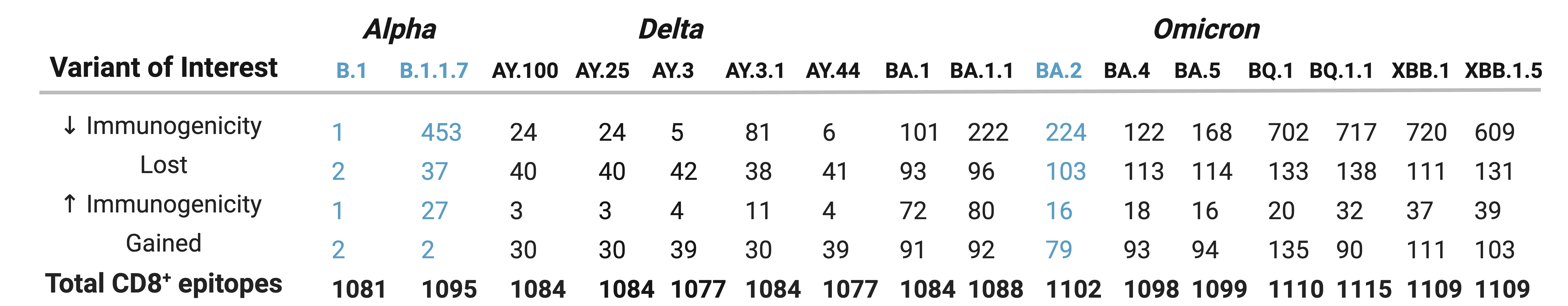


Fig. S4. CD8+ spike epitope differences of variants from Ancestral Wuhan sequence.

Epitope differences between variants of interest and ancestral Wuhan strain from predicted binding generated with IEDB’s TepiTool’s (35) panel for the 27 most common HLA class I alleles. Lost epitopes are those present in the Wuhan strain but absent in the new variant, whereas gained epitopes are absent in Wuhan but present in the new variant. This figure was adapted from (28) and generated on Biorender.

Table S1. Adjusted odds ratio of demographic effects.

Demographic effects were adjusted for the other demographics and variants. For example, the female (versus male) effect was adjusted for race, age, and variant. The adjustment matched cases on variant and used the other terms as covariants.

| ***Risk Factor*** | ***Risk factor present (n=)*** | ***Risk factor absent (n=)*** | ***Odds ratio*** | ***Lower 95% CI*** | ***Upper 95% CI*** | ***P value*** |
| --- | --- | --- | --- | --- | --- | --- |
| **Female** | 1636 | 1287 | 1.317 | 1.145 | 1.514 | 0.000 |
| **African American** | 1044 | 1877 | 0.956 | 0.805 | 1.136 | 0.611 |
| **Hispanic** | 162 | 2677 | 0.952 | 0.615 | 1.474 | 0.825 |
| **Older age (+65)** | 531 | 2376 | 1.947 | 1.523 | 2.489 | 0.000 |

Table S2.Vaccination Demographics

Vaccinated patients were significantly more likely to be older and female. Additionally, vaccinated patients had significantly greater representation of participants over 65 years old (17.6% vs 5.8%, p < 0.0001) and women over 50 years (26.2% vs 10.7%, p < 0.0001).

| **Age Demographics** | | **Age** ± **Standard Deviation or Count (Percentage)** | **Long COVID** | | **T/Chi-square test** |
| --- | --- | --- | --- | --- | --- |
|  |  |  | Yes | No | **P-values** |
| **Overall Count** | | 3,090 (100) | 1554 (50.29) | 1536 (49.71) |  |
| **Overall Age** | | 39.53 ±20.24 | 43.09 ±20.30 | 35.94 ±19.52 | 0.0054 |
|  | Female Age | 40.20 ± 20.00 | 43.28 ± 19.95 | 36.75 ± 19.50 | < 0.0001 |
|  | Male Age | 38.67 ± 20.51 | 42.81 ± 20.83 | 35.02 ± 19.52 | < 0.0001 |
|  | **+50 years old** | 1031 (33.3) | 642 (62.2) | 389 (37.7) |  |
|  | **+50 female** | 591 (19.1) | 366 (61.9) | 225 (38.1) |  |
|  | **+65 years old** | 378 (12.2) | 250 (66.1) | 128 (33.9) |  |
|  | **+65 female** | 225 (7.3) | 148 (65.8) | 77 (34.2) |  |
| **Vaccinated Count** | | 1683 (54) | 1007 (59.8) | 676 (40.2) |  |
| **Vaccinated Age** | | 45.87 ± 18.80 | 47.95 ± 18.61 | 42.76 ± 18.67 | < 0.0001 |
|  | Female Age | 45.36 ± 18.76 | 46.99 ± 18.87 | 42.86 ± 18.34 | 0.0005 |
|  | Male Age | 46.68 ± 18.9 | 49.56 ± 18.09 | 42.61 ± 19.21 | < 0.0001 |
|  | **+50 years old** | 747 (44.4) | 500 (66.9) | 247 (33.1) |  |
|  | **+50 female** | 441 (26.2) | 289 (65.5) | 152 (34.5) |  |
|  | **+65 years old** | 296 (17.6) | 207 (69.9) | 89 (30.1) |  |
|  | **+65 female** | 180 (10.7) | 123 (68.3) | 57 (31.7) |  |
| **Unvaccinated Count** | | 1407 (45.5) | 547 (38.9) | 860 (61.1) |  |
| **Unvaccinated Age** | | 31.96 ± 19.27 | 34.14 ± 20.27 | 30.58 ± 18.49 | 0.0007 |
|  | Female Age | 32.51 ± 19.31 | 35.18 ± 19.88 | 30.62 ± 18.70 | 0.0021 |
|  | Male Age | 31.42 ± 19.2 | 32.97 ± 20.67 | 30.54 ± 18.31 | 0.1054 |
|  | **+50 years old** | 284 (20.2) | 142 (50) | 142 (50) |  |
|  | **+50 female** | 150 (10.7) | 77 (51.3) | 73 (48.7) |  |
|  | **+65 years old** | 82 (5.8) | 43 (52.4) | 39 (47.6) |  |
|  | **+65 female** | 45 (3.2) | 25 (55.6) | 20 (44.4) |  |

Table S3. Odds ratio by PANGO variant.

The odds ratios compare odds of developing Long COVID among those with a particular variant to the odds of developing Long COVID for all other variants combined. The odds ratios are adjusted, via stratification, for age, gender, and race. An odds ratio > 1.0 implies the PANGO variant had higher adjusted odds of developing Long COVID than did the combination of all other variants. In creating each combination of other variants, each of the other variants contributed to the combination in proportion to its sample size.

| ***PANGO variant*** | ***Pandemic Era*** | ***Variant sequences (n=)*** | ***Comparison variants sequences (n=)*** | ***Adjusted Odds Ratio (95% CI)*** | ***P value*** |
| --- | --- | --- | --- | --- | --- |
| B.1 | Pre-Alpha | 244 | 2680 | 1.955 (1.359 to 2.812) | **0.000** |
| B.1.1.7 | Alpha | 75 | 2849 | 2.396 (1.229 to 4.672) | **0.010** |
| AY.100 | Delta | 62 | 2862 | 1.519 (0.746 to 3.092) | 0.249 |
| AY.103 | Delta | 73 | 2851 | 0.961 (0.502 to 1.838) | 0.903 |
| AY.119 | Delta | 25 | 2899 | 0.790 (0.260 to 2.401) | 0.678 |
| AY.25 | Delta | 380 | 2544 | 1.142 (0.859 to 1.517) | 0.362 |
| AY.26 | Delta | 82 | 2842 | 1.366 (0.739 to 2.525) | 0.319 |
| AY.3 | Delta | 234 | 2690 | 1.182 (0.822 to 1.699) | 0.367 |
| AY.39 | Delta | 22 | 2902 | 0.909 (0.277 to 2.988) | 0.875 |
| AY.44 | Delta | 106 | 2818 | 0.997 (0.582 to 1.709) | 0.992 |
| BA.1 | Omicron | 956 | 1968 | 0.839 (0.701 to 1.005) | 0.056 |
| BA.2 | Omicron | 309 | 2615 | 0.599 (0.436 to 0.824) | **0.002** |
| BA.4 | Omicron | 68 | 2856 | 0.700 (0.356 to 1.375) | 0.301 |
| BA.5 | Omicron | 258 | 2666 | 0.732 (0.518 to 1.035) | 0.078 |
| BQ.1 | Omicron | 30 | 2894 | 0.743 (0.269 to 2.050) | 0.566 |
